# How various design decisions on matching individuals in relationships affect the outcomes of microsimulations of sexually transmitted infection epidemics

**DOI:** 10.1371/journal.pone.0202516

**Published:** 2018-08-29

**Authors:** Nathan Geffen, Stefan Michael Scholz

**Affiliations:** 1 Centre for Social Science Research, University of Cape Town, Cape Town, South Africa; 2 Department of Public Health, University of Bielefeld, Bielefeld, Germany; 3 Center for Health Economics Research Hannover (CHERH), Leibniz-University Hannover, Hannover, Germany; Laboratoire National de Santé, LUXEMBOURG

## Abstract

Microsimulations are increasingly used to estimate the prevalence of sexually transmitted infections (STIs). These models consist of agents which represent a sexually active population. Matching agents into sexual relationships is computationally intensive and presents modellers with difficult design decisions: how to select which partnerships between agents break up, which agents enter a partnership market, and how to pair agents in the partnership market. The aim of this study was to analyse the effect of these design decisions on STI prevalence. We compared two strategies for selecting which agents enter a daily partnership market and which agent partnerships break up: random selection in which agents are treated homogenously versus selection based on data from a large German longitudinal data set that accounts for sex, sexual orientation and age heterogeneity. We also coupled each of these strategies with one of several recently described algorithms for pairing agents and compared their speed and outcomes. Additional design choices were also considered, such as the number of agents used in the model, increasing the heterogeneity of agents’ sexual behaviour, and the proportion of relationships which are casual sex encounters. Approaches which account for agent heterogeneity estimated lower prevalence than less sophisticated approaches which treat agents homogeneously. Also, in simulations with non-random pairing of agents, as the risk of infection increased, incidence declined as the number of agents increased. Our algorithms facilitate the execution of thousands of simulations with large numbers of agents quickly. Fast pair-matching algorithms provide a practical way for microsimulation modellers to account for varying sexual behaviour within the population they are studying. For STIs with high infection rates modellers may need to experiment with different population sizes.

## Introduction

Microsimulations or agent-based models (ABMs) are increasingly used to simulate incidence and prevalence of STIs as well as to identify the costs and benefits of strategies to contain them. Diseases modelled using this approach include HIV [1–3], syphilis [4], gonorrhoea [5], HPV [6], herpes, chlamydia and trichomoniasis [7]. The popularity of such stochastic microsimulations may lie in the easier implementation of complex, heterogeneous sexual behaviour when compared to traditional ordinary differential equation (ODE) models.

However, there are many design decisions which must be made for microsimulations, with respect to sexual behaviour modelling. For example, which agents should be considered for relationships (i.e. placed in a partnership market), how agents in the partnership market should be paired with one another, and which relationships should terminate (breakups). These decisions may be informed by agent characteristics, such as age, sex and sexual orientation, but also by individual variations in average behaviour, e.g. sexual risk taking and propensity to remain in relationships. (This has similarities with regression analysis for longitudinal data: Some variation of the dependent variable can be explained by observable, group-level characteristics like age, sex, and sexual orientation., but adding a random effect for unobservable, individual characteristics may explain additional variation.)

It is well understood how the design decisions for ODE models affect outcomes [8], including for specific diseases [9], but much less so for STI microsimulations. The aim of our research is to fill this gap and to understand how the above-mentioned design decisions affect estimates of disease incidence and prevalence. Specifically we want to analyse the effect of different algorithms for matching and unmatching agents and their interaction with (I) the probability of transmission, (II) the size of the model population, and (III) heterogeneity in agent behavior.

To do this we present a microsimulation model with behaviour based on data drawn from the German population, and which simulates the spread of a generic, fictitious STI in a fixed cohort. To isolate the effects of different approaches to partner matching and breakups, and to ensure that prevalence is always cumulative incidence, the STI has no recovery rate, births, deaths or migration. Thus, the model is an Susceptible-Infected (SI) model. The prevalence estimated at the end of our simulations is a function of the risk of infection in serodiscordant partnerships, the number of partnerships over time, and the distribution of partnerships over time. The last of these is especially affected by how the partnership market is chosen, the algorithm that pairs agents in the partnership market, and how breakups are modelled. Moreover the number and distribution of partnerships can also be affected by the likelihood of casual sex encounters (modelled as partnerships lasting one day in our simulations), and the heterogeneity of agent sexual behaviour. (Note on terminology: We use *microsimulation* and *agent based model* synonymously. A single execution of a microsimulation model, usually but not always 10 years in our experiments, is called a *simulation* or *run*. A *time step* is a single iteration of a simulation, which happens to always be one day in the experiments described here. A *partnership market* is a subset of agents on each day that must be *paired* into relationships. We use *partnership*, *relationship* and *pair* synonymously. The terms *breakup* and *unmatch* describe the termination of a relationship.) Appendix 1 discusses the implementation details of the microsimulation with respect to the necessary speed of the simulation runs.

## Materials and methods

### General structure of the model

We implement a discrete time step microsimulation which has the structure of Algorithm 1. (The code is available under the GNU General Public License Version 3. It is written in C++ and available on Github at: https://github.com/nathangeffen/faststi.)

Users can specify the number of agents, the time period for which to run the simulation, and the daily risk of infection for a seronegative agent in a serodiscordant partnership. This risk is specified based on the sex of the uninfected agent and whether it is an opposite- or same-sex relationship. The time step of the model is one day in all the experiments described in this paper. On each day the following series of events is executed on all or a subset of agents:

**Age** Each agent becomes a day older.
**Infect** Each uninfected agent in a relationship with an infected one may become infected.
**Breakup** All the relationships are traversed and some of them are terminated.
**Select** A subset of unpaired agents is selected to enter a partnership market. If there is an odd number of agents in the partnership market, a randomly chosen one is removed.
**Match** Agents in the partnership market are matched with each other.

An exception to this is when the model is running in *stabilisation mode*. This is sometimes done at the beginning of a simulation to stabilise the number of daily breakups and pairings. During this phase neither the *age nor* infect events are executed.

**Algorithm 1** Structure of a discrete microsimulation from [10]

1: **for** each time step **do**

2:  **for** each event *e* **do**

3:   **for** each agent *a* **do**

4:    **if** *e* has to be applied to *a* **then**

5:     Apply *e* to *a*

6:    **end if**

7:   **end for**

8:  **end for**

9: **end for**

Each agent has a sex, sexual orientation and age which can be used to identify the corresponding characteristics of its preferred partner. An agent’s sex and age determines its daily risk of entering the partnership market if it is single, or breaking up if it is in a relationship.

### Population characteristics and agent behavior

All single agents are initialized to between 12 and 50 years old, proportionate to the German population. The vast majority of agents in partnerships are initialised to between 12 and 50. (The initialization routine sometimes creates agents outside this age range to be partners of agents in the 12 to 50 age range. All agents are included in analysis, irrespective of their age. As agents age, their sexual behaviour is updated.)

The behavior of the agents includes probabilities for breakups, entering the partnership market for long-term relationships and casual sex encounters. To model heterogeneous versus homogeneous behavior, two different strategies, called RANDOM and DATA, were implemented.

- The RANDOM strategy contains no heterogeneous behavior as it randomly selects a set of partnerships to break up and a set of agents to enter the partnership market. On average the same number of agents break up as enter the partnership market each day.
- The DATA strategy estimates daily, group-level (i.e., age- and sex-specific) probabilities for breakups, partnership formation and casual sex contacts from *The German Family Panel* (pairfam) [11, 12]. This longitudinal dataset provides information about the complete relationship history of the study participants before the beginning of the survey as well as during the survey period. For the latter, information about the frequency of sexual intercourse is provided for the three months preceding the interview date of each wave of the survey. To determine the probabilities of breakups and entering a new long-term relationship, the beginnings and ends of partnerships are extracted for each study participant for all the relationships and this data is summarised for all study participants by each age year. The probabilities are then calculated by dividing the number of breakups by the number of relationships at each age and the number of new relationships by the number of single persons at each age. To estimate the probability of casual sex contacts the number of sexual intercourses in the three months before the survey are converted to daily probabilities for people who indicated that they had not been in a relationship during that period. When calculating the risk of a breakup, the sum of the agents’ breakup probabilities are averaged.
  As men-who-have-sex-with-men (MSM) or women-who-have-sex-with-women (WSW) are not represented well in the data set, the probabilities have been estimated irrespective of sexual orientation. The estimates of the age-specific model parameters can be found in Appendix 2. Agents older than the highest age—50 years—for which data is available, are treated the same as 50-year-olds.

By default the DATA strategy models group-level heterogeneity: differences in sexual behaviour by sex and age. The implementation is as follows: Consider how an agent enters the partnership market (i.e. the Select event). A uniform random number between 0 and 1 is generated. If the agent’s probability, *p*, of entering the partnership market, calculated based on sex and age, is greater than this number, the agent enters the partnership market. Analogous mechanisms are used for determining whether the agent will have a casual relationship, and whether a relationship breaks up (in this case the mean of the probabilities of the two agents is used).

To additionally model unobservable, individual-level heterogeneity, *p* is multiplied by a factor with normal distribution of mean 1 and standard deviation 0.3, but with a maximum value of *p* = 1. This factor is set individually for each agent at the beginning of the simulation and does not change over time. There are actually three such factors: one for entering the partnership market in search of a casual sex relationship, one for entering the partnership market for a non-casual sex relationship, and one for breaking up, thereby generating a wide variety of individual behaviour. However, except for one set of experiments, this individual-level heterogeneity is deactivated.

While some of the parameters chosen to do these simulations are arbitrary (such as the standard deviation of 0.3 in the above paragraph), our aim is proof of concept. Modelling specific diseases will require choosing appropriate parameters informed by data.

### Matching procedures and algorithms

By default the simulation matches all agents in the partnership market. If the number of agents in the market is odd, one is randomly removed. The matching algorithms have been described and their performances analysed in [10]. They depend on the existence of a distance function that measures the suitability of two agents for matching based on sex, sexual orientation and age according to the distribution of relationships in the population. The smaller the distance the more suitable are the agents for matching. All but one of the algorithms attempt to minimise the sum of the distances of all matches.

Using distance to measure the suitability of a match has the following advantages: (1) the algorithms can be kept generic with domain specific details confined to the distance function, (2) category mismatches (such as agents with different sexual orientations) can be dealt with by the distance function returning very large values rendering such matches unlikely or even impossible if the penalization is higher than a maximal distance threshold for matching, and (3) it provides a measurement for comparing how closely algorithms estimate the underlying distribution. Algorithm 2 provides the distance function we used.

**Algorithm 2** Distance function used in simulations

**Parameters:**

*a*, *b***: agents between whom to measure distance**

**Return value: real number that determines how likely a partnership is between** *a* **and** *b* **according to the distribution of partnerships in the population being studied.**

**0 is a good match, while 50 or more is a poor one.**

**A modification in some simulations we ran was to remove the previous partnership penalty.**

1: **function** distance(*a*, *b*)

2:  *ageProb* ← lookup probability of matching *a* with *b* based on ages.

3:  *agePenalty* ← (1 − *ageProb*) * 50

4:  **if** mismatch on sex based on sexual orientation **then**

5:   *orientationPenalty* ← 50

6:  **end if**

7:  **if** *a* and *b* have been partners previously **then**

8:   *prevPenalty* ← 50

9:  **end if**

10:  **Return** *agePenalty* + *orientationPenalty* + *PrevPenalty*

11: **end function**

Two algorithms serve as upper and lower boundaries of the quality of matches. *Random-Pair Matching* (RPM), in which agents in the partnership market are paired randomly, sets the lower limit on quality. The average distance between paired agents in the partnership market that the other algorithms generate should be much smaller than that of RPM.

On the other end of the scale, the Blossom algorithm, first described by [13], finds the minimum sum of the distances of pairs of vertices in a graph. We use it by first generating a fully connected undirected graph in which each vertex represents an agent in the partnership market and each edge represents the distance between two agents. Under the assumptions that (1) the distance functions contains relevant components influencing the partner selection, and (2) that the quality of the data sources used to inform the distance function, the Blossom algorithm finds the theoretically closest set of pairs to the distribution of relationships in the population being studied.

The problem with Blossom is that it is impractically slow when there are a large number of agents in the partnership market, even when using a highly optimised recent implementation called Blossom V [14]. The time to create the graph increases quadratically with the number of agents in the partnership market, and the time for the Blossom V algorithm increases approximately cubicly with the number of agents in the partnership market. Generally, algorithms in a simulation whose execution time increases more than linearithmically (*n* log *n*) with the number of agents are impractical if modellers wish to do sensitivity analysis, calibrate parameters, or build confidence intervals (see Appendix 1). Furthermore it is not a stochastic algorithm which is often a desired feature of pair-matching.

Between the high and low precision of Blossom and RPM, respectively, are algorithms that approximate the minimum sum of distances. With the exception of the Cluster Shuffle Pair Matching (CSPM) algorithm, all algorithms store the agents randomly in an array to avoid systematic bias in the matching. We also used the following matching algorithms in our analyses:

- *Random K pair matching* (RKPM) is similar to RPM. For each agent *a* in the partnership market that still needs to be matched, it examines up to *k* adjacent neighbouring agents in the partnership market—where *k* is a user-defined constant positive integer that is usually much smaller than the number of agents in the partnership market—and matches *a* with the agent with the lowest distance to it. RPM is essentially RKPM with *k* = 1.
- *Brute-Force pair matching* (BFPM) is similar to RKPM except that k is set to a value equal to or greater than the maximum number of agents in the partnership market. This means that for each agent *a* that still needs to be matched, it will be partnered with the remaining unmatched agent that has the shortest distance to it.
- *Cluster Shuffle Pair Matching* (CSPM) relies on the existence of a cluster function as described by [10]. It sorts the agents by the value returned by the cluster function. The sorted agents are divided into a user-specified number of clusters. Each cluster is then shuffled to introduce stochasticism. Next, as with RKPM, for each unmatched agent *a*, it examines the *k* adjacent neighbouring agents in the partnership market, choosing the one with the lowest distance to *a*. Since it is more complex than the above two algorithms, pseudocode for the CSPM algorithm is presented in Algorithm 3.

**Algorithm 3** Cluster shuffle pair-matching (CSPM)

**Parameters:**

*Agents*, **an array of agents, with subscripts** 0..*n* − 1, **where** *n* **is the number of agents. For simplicity assume** *n* **is even.**

*c*, **the number of clusters to divide the agents into. For simplicity assume** *c* **divides into** *n*.

*k*, **the number of adjacent agents to consider when finding a suitable partner.**

1: **function** clusterShuffleMatch(*Agents*, *c*, *k*)

2:  **for** each agent, *a*, in *Agents* **do**

3:   *a*.*weight* ← *cluster*(*a*)

4:  **end for**

5:  sort *Agents* by weight

6:  *clusterSize* ← *n*/*c*

7:  *i* ← 0

8:  **for** each cluster **do**

9:   *first* ← *i* * *clusterSize*

10:   *last* ← *first* + *clusterSize*

11:   shuffle *Agents*[*first*…*last* − 1]       ▹ to introduce stochasticism

12:   *i* ← *i* + 1

13:  **end for**

14:  **for** each unmatched agent *a* in *Agents* **do**

15:   *best* ← ∞

16:   **for** each unmatched agent *b* in one of up to *k* positions in the array after *a* **do**

17:    *d* ← *distance*(*a*, *b*)

18:    **if** *d* < *best* **then**

19:     *best* ← *d*

20:     *bestPartner* ← *b*

21:    **end if**

22:   **end for**

23:   Make *a* and *bestPartner* partners

24:  **end for**

25: **end function**

[10] discusses the selection of values of *k* in the CSPM and RKPM algorithms, and the number of clusters in the CSPM algorithm. In this work we varied *k* between 30 and 300, and we varied the number of clusters between 10 and 100, depending on the number of agents in the simulation’s population.

In summary, a simulation therefore has a breakup and partnership market strategy (DATA vs RANDOM) coupled with a pair-matching algorithm (Blossom vs BFPM vs CSPM vs RKPM vs RPM). (We use “strategy” to describe the *Select* and *Breakup* events, and “algorithm” for the *Match* event so as to keep RANDOM and DATA differentiated from Blossom, BFPM, CSPM, RKPM and RPM in the reader’s mind.)

The DATA strategy coupled with Blossom, BFPM, CSPM or RKPM accounts for the differences between agents, while the RANDOM strategy coupled with RPM treats all agents equally. DATA coupled with RPM, and RANDOM coupled with Blossom, BFPM, CSPM or RKPM represent compromises between respecting and ignoring agents’ characteristics.

Accounting for group-specific differences in sexual behaviour makes matches more representative of the population being studied, and prevents the model from overestimating the spread of the STI. However, all the algorithms generate at least some poor matches, i.e. matching agents across vastly different age groups or with differing sexual orientations. These “mismatches”, provided they are not too frequent, actually assist the simulation by ensuring the STIs eventually cross into different subgroups. It is possible to set the model to ignore poor matches, but this comes with severe disadvantages: (a) the number of matches, especially with the RANDOM strategy or RPM algorithm, would be too few, and (b) we would in essence have several entirely independent subgroups such as MSM and men who have sex with women (MSW) having no effect on each other, which is not realistic and defeats the purpose of using a microsimulation as opposed to an ODE model with multiple compartments.

### Simulation set-up

#### General set-up

Our aim is to show the qualitative effects of design decisions on prevalence. Hence, the results presented here should be seen as illustrative; their precise impact will be a function of the particular disease, setting and time being modelled.

In these experiments, the group of MSM between the ages of 15 and 20 are initiated to an infected state in the initial population. The generic STI spreads out from this group across the population. This results in about 1 in 1,000 agents being infected in the initial population. (We also re-ran several simulations which, instead, distributed the initial infections across various demographic groups. There was no qualitative difference in the results.)

#### Experiment set-ups

To determine the effect of the different pair-matching algorithms on prevalence under different risk of transmission scenarios (research question I), we ran the algorithms in simulations of 20,000 and one million agents for ten years with a time step of one day. It was only feasible to execute the Blossom algorithm with 20,000 agents as it is simply too slow on a population of 1 million agents. For this experiment we chose different scenarios of the transmission probability per contact (low, medium and high risk of infection as given in Table 1) with all other model parameters held constant. Since the model is stochastic, we generally repeated each simulation 30 times to build mean final prevalence and confidence intervals. From our experience in HIV modeling, a higher infectiousness for males was implicitly assumed.

**Table 1:** Daily risk of infection for susceptible agent in a discordant relationship.

	Risk Scenario					
	Low		Medium		High	
	Male	Female	Male	Female	Male	Female
Male	0.002	0.001	0.02	0.01	0.2	0.1
Female	0.002	0.001	0.02	0.01	0.2	0.1

A second experiment was set up to further analyse the effect of the different algorithms in combination with various different population sizes, ranging from from 10,000 to 1 million agents, answering research question II.

To analyse the effect of agent heterogeneity a two-step experiment was conducted (research question III). First, the effect of group-level heterogeneity was analysed by comparing the RANDOM and DATA strategy for agent behavior. To stabilise the number of daily breakups and partnership market entrants in the DATA strategy, the stabilisation period was set to 60 days (i.e. no ageing or infections occur in this period). To avoid bias in our comparison of strategies, the stabilisation period was also run with the RANDOM strategy.

### Relevance to various STIs

Table 2 summarises estimated transmission probabilities for various STIs from the literature for comparison to the different transmission scenarios used in our model. The way transmission is modelled varies considerably between models and ranges from modelling a single transmission route in a sexual contact to the transmission per partnership. Transmission probabilites are therefore not easily comparable, but must be seen with respect to their unit of reference. As our model uses the transmission probabilities per day, the above scenarios are easily comparable for the estimates for gonorrhea and chlamydia used by Kretschmar et al. [15].

**Table 2:** Probabilities of infection for different STIs. (URAI = Unprotected, receptive penile-anal intercourse; UIAI = Unprotected, insertive penile-anal intercourse; MtoF = male to female transmission; FtoM = female to male transmission; ^1^act/transmission route not further specified.)

STI	Unit	Tranmission probability	Comment	Source
HIV	act	0.014 [95%CI 0.002;0.025]	URAI	[16]
	partner	0.404 [95%CI 0.060;0.749]	URAI	
	partner	0.217 [95%CI 0.160;0.429]	UIAI	
Syphilis	act	0.014	UAI	[4]
	partner	0.627		[17]
HPV	act	0.400 (range 0.050–1.000)	Simulated^1^	[18]
	partner	0.270 [95%CI 0.210;0.350]	MtoF^1^	[19]
	partner	0.310 [95%CI 0.240;0.400]	FtoM^1^	[19]
Gonorrhea	day	0.150/0.600 (steady/casual)	MtoF^1^	[15]
	day	0.063/0.250 (steady/casual)	FtoM^1^	
Chlamydia	day	0.039/0.154 (steady/casual)	MtoF^1^	[15]
	day	0.305/0.122 (steady/casual)	FtoM	

Unfortunately, it is not possible to present the transmission probabilities on the same scale because not all sources stated the types and frequencies of transmission routes per sexual contact, the number of sexual contacts per partnership, or the duration of partnerships.

### Ethics

This article is based on secondary analyses. Compliance with ethical standards for German social research and data protection laws were secured throughout by the project team of the pairfam study. Informed consent was obtained from all individual participants included in the study by the professional interviewer at the beginning of each interview.

## Results

### Effect of pair matching algorithms and transmission probability

Table 3 shows the results of the model using the different matching algorithms. In the low-risk scenario, there was no significant difference in prevalence by algorithm, irrespective of whether a small (20,000) or large (1 million) number of agents was used. But as the risk increased RKPM, CSPM, BFPM and Blossom showed a trend towards lower prevalence compared to RPM, and this trend was significant for simulations with 1 million agents. Moreover, all the algorithms, except RPM, calculated lower prevalence with a higher number of agents. The higher the risk of transmission the more sensitive the final prevalence was to the number of agents in the simulation. Also, as expected, the confidence intervals were narrower for 1 million versus 20,000 agents for all algorithms.

**Table 3:** Prevalence after 10 years of low, medium and high infection risk scenarios for pair-matching algorithms, sorted by prevalence of high risk scenario. Each entry in the Low, Medium and High columns is the mean and 95% confidence interval of 30 runs.

N	Algorithm	Infection risk scenario		
		Low	Medium	High
20,000	RPM	0.3% [0.2;0.4]	1.1% [0.7;1.8]	50.8% [47.9;52.0]
	RKPM	0.3% [0.2;0.4]	1.1% [0.8;1.5]	48.9% [46.5;51.6]
	BFPM	0.3% [0.2;0.4]	1.0% [0.6;1.4]	49.3% [47.7;51.4]
	CSPM	0.4% [0.2;0.5]	1.0% [0.5;1.5]	48.2% [45.3;49.8]
	BLOSSOM	0.3% [0.2;0.4]	1.0% [0.8;1.4]	47.7% [46.5;48.9]
1,000,000	RPM	0.3% [0.3;0.3]	1.1% [1.0;1.1]	51.1% [50.9;51.3]
	RKPM	0.3% [0.3;0.3]	0.8% [0.8;0.8]	46.2% [46.0;46.5]
	BFPM	0.3% [0.2;0.3]	0.5% [0.4;0.5]	43.8% [43.4;44.4]
	CSPM	0.3% [0.3;0.3]	0.8% [0.7;0.8]	37.7% [36.3;39.2]
	Blossom	NA	NA	NA

Except for RPM, all the pair-matching algorithms in the high-risk scenario—and to a lesser extent with the medium-risk scenario—resulted in lower prevalence in the population with 1 million agents. By contrast, RPM generated the same prevalence, irrespective of the population size. As the equilibrium of an STI model without birth is reached as soon as all persons are infected, these results suggest, that the equilibrium in our model is reached faster in scenarios of higher infectiousness, with smaller population size and with algorithms that match agents randomly.

Blossom is too slow to run with 1 million agents, but with 100,000 agents the mean final prevalence over 12 runs was 42%, compared to 47.7% with 20,000 agents (see Table 4). Again, this might suggest that the time to equilibrium increases with population size and the more complicated matching algorithms.

**Table 4:** Comparison of prevalence for CSPM against Blossom after 10 years for three different population sizes. Each entry in the CSPM and Blossom columns is the mean of 12 runs.

Population	CSPM	Blossom
10,000	47.9%	48.7%
50,000	45.5%	44.3%
100,000	43.9%	42.0%

### Effect of population size

**Fig 1:**
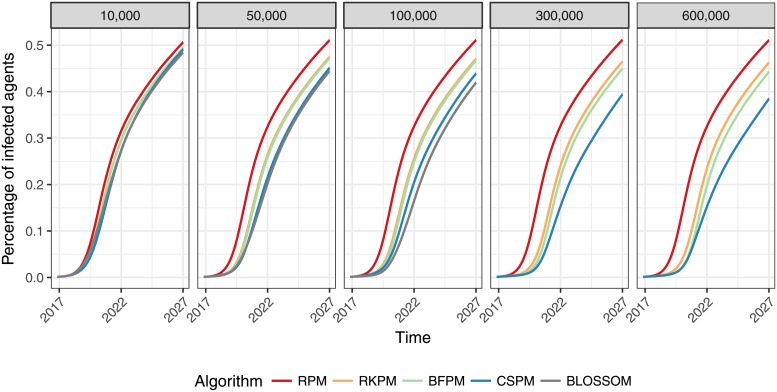
Disease prevalence by population. Mean disease prevalence after 10 years of 30 simulation runs for different population sizes of 10,000, to 600,000 agents.

To follow up the different results for the different population sizes, we ran further simulations in the high-transmission risk scenario for 10,000, 50,000 and 100,000 agents for all algorithms, and 300,000 and 600,000 agents for all algorithms except Blossom. As Fig 1 shows, the simulations reveal a pattern of lower prevalence after 10 years for a higher number of agents and for the more complex algorithms.

**Fig 2:**
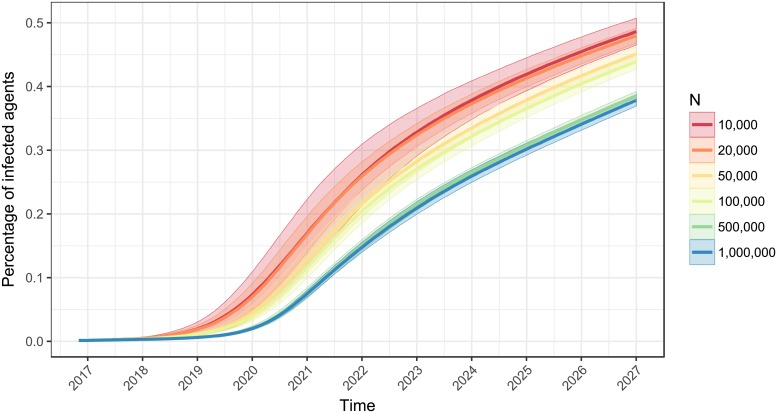
DATA/CSPM simulations for different population sizes. DATA/CSPM simulations run for ten years (3,650 days) on 20,000, 300,000, 500,000 and 1 million agents. The lower prevalence with higher number of agents appears to be a consequence of the longer time that the STI takes to begin growing rapidly in its early stage.

We also examined the behavior of the CSPM algorithm for different population sizes (10,000, 20,000, 50,000, 100,000, 500,000 and 1 million) to explore if the effect of decreasing prevalence with increasing number of agents tapers off. Fig 2 depicts this visually, showing that reduced prevalence is much greater moving from 100,000 to 500,000 agents, than from 500,000 to 1 million agents.

Running the medium-risk scenario for long enough demonstrated that the effect of declining prevalence as the number of agents increases that we saw with the high risk scenario was still present, but took longer to be as noticeable. Average final prevalence for 10,000 agents for a 100 years using CSPM was 81.7% [95%CI: 65.4;90.2 over 200 runs] for 10,000 agents versus 74.1% [95%CI 73.2;74.9 over 16 runs] for 1 million agents.

### Heterogeneity of agents entering the partnership market and breaking up

#### Effect of group-level heterogeneity

We used a similar methodology to compare the DATA and RANDOM strategies (using only CSPM and RPM as the pair-matching algorithms). The daily number of breakups and agents entering the partnership market in the RANDOM strategy was set to closely match (less than 0.2% difference) the daily average for the DATA strategy. The results are presented in Table 5.

**Table 5:** Prevalence after 10 years of low, medium and high infection risk scenarios for breakup and partnership market strategies, sorted by prevalence of high risk scenario. Each entry in the Low, Mean and High columns is the mean and 95% confidence interval of 30 runs.

Strategy	Algorithm	#Agents	Infection risk scenario		
			Low	Medium	High
DATA	CSPM	1,000,000	0.3% [0.3;0.3]	0.8% [0.7;0.8]	37.7% [36.3;39.2]
		20,000	0.4% [0.2;0.5]	1.0% [0.5;1.5]	48.2% [45.3;49.8]
RANDOM	CSPM	1,000,000	0.9% [0.8;0.9]	39.0% [37.8;40.2]	81.7% [78.3;88.1]
		20,000	3.2% [2.1;4.0]	100% [99.9;100]	100% [100;100]
	RPM	1,000,000	3.5% [2.9;3.9]	100% [100;100]	100% [100;100]
		20,000	3.4% [1.3;4.8]	100% [100;100]	100% [100;100]

The DATA strategy coupled with CSPM resulted in the lowest estimates of prevalence in all risk scenarios, followed by RANDOM coupled with CSPM. The approach that treats agents entirely equal, RANDOM coupled with RPM, estimated the highest prevalence.

#### Effect of individual-level heterogeneity

To test individual-level heterogeneity we ran 30 simulations each with DATA coupled with CSPM for 10,000, 50,000, 100,000, and 500,000 agents over 10 years with the high risk scenario with and without individual-level heterogeneity. The results are presented in Table 6. There were no significant differences in mean final prevalence. Confidence intervals were also roughly the same width.

**Table 6:** Mean prevalence and 95% confidence interval over 30 runs comparing group- (age, sex and sexual orientation) versus individual-level heterogeneity (age, sex and sexual orientation modified by factors set for each agent).

Agents	Group	Individual
10,000	48.5% [45.8;51.0]	47.8% [42.2;51.9]
50,000	45.9% [44.5;47.4]	46.3% [44.4;48.1]
100,000	43.9% [41.6;45.5]	44.4% [43.1;45.8]
500,000	38.6% [37.5;39.7]	40.2% [38.5;41.9]

Supplementing group-level heterogeneity by individual-level in this way and to this extent does not appear to affect *overall* prevalence. However, we do not rule out the possibility that a more in-depth analysis may revise this finding.

### Effects of other design decisions

We explored several other issues that may effect incidence and prevalence estimates.

#### Changing the number of partnerships

In the default data set we used, the average number of partners per agent per year was 2.9. The output of the model showed that 98% of all these are casual interactions. To see the effect of reducing partnerships across the model, for the DATA strategy coupled with CSPM, we compared the effect on prevalence of reducing the casual partnerships by 50%, 75% and 90%.

Table 7 presents the results of these simulations, showing that even after massively reducing the partnerships the prevalence still declines with an increase in the number of agents for the medium- and high-risk scenarios. However, the effect becomes less pronounced as the number of partnerships declines.

**Table 7:** Results of simulations with reduced number of casual partnerships. Each entry in the Low, Medium and High is the mean and 95% confidence interval over 30 runs.

# Agents	% of default	Infection risk scenario		
		Low	Medium	High
20,000	50	0.4% [0.2;0.5]	0.7% [0.4;1.1]	29% [24.1;32.8]
	25	0.3% [0.2;0.4]	0.6% [0.4;0.8]	6.1% [3.8;8.1]
	10	0.3% [0.2;0.4]	0.5% [0.3;0.7]	1.1% [0.6;1.6]
1,000,000	50	0.3% [0.3;0.3]	0.5% [0.5;0.6]	16.7% [16.1;17.7]
	25	0.3% [0.3;0.3]	0.5% [0.4;0.5]	2.8% [2.6;3]
	10	0.3% [0.3;0.3]	0.4% [0.4;0.4]	0.8% [0.8;0.9]

#### Discouraging previous partnerships

A dilemma we had was how to deal with a potential new partnership between agents who had previously been partners. We implemented two variations of the distance function, one that keeps track of all partnerships and penalises potential pairings between agents who have previously been in a relationship, and one that does not keep track of partnerships at all. We found no meaningful difference in results using these two methods.

There are practical implementation consequences of this finding. Our largest simulations (40 million agents, see Appendix 1) use a large amount of memory. The number of simulations we could run in parallel was limited by the available memory on our machine. The largest data structure by far in our simulations, despite extensive optimisation, is the one that keeps track of previous partnerships. With the finding that penalising previous partnerships made no difference to the results, this data structure could be disabled on large simulations, allowing more simulations to be run in parallel.

## Discussion

The aim of our paper was to explore the influence of model design decisions on the results of epidemiological individual based models with the focus on the choice of matching algorithm and their interactions with transmission probability, population size and population heterogeneity. Our experiments found the following:

- Random matching of agents in an STI microsimulation leads to higher infection incidence than non-random matching. This is unsurprising and a consequence of not accounting for differences in sexual behaviour of agents.
  With less randomness in pair-matching, the STI is more confined to subgroups that have riskier sexual behaviour profiles. When mating and breaking up randomly (i.e. treating all agents equally), the infection spreads uniformly through the population.
- Confidence intervals around the prevalence estimates after 10 years of simulation narrow as the number of agents in the population increases.
- The incidence and prevalence that microsimulations using sophisticated pair-matching algorithms estimate are sensitive to the number of agents in the model. As the risk of infection for the sero-negative partner in a sero-discordant partnership increases, or as the frequency of partnership formation and breakups in the population increases, the more sensitive to the number of agents the model becomes.
- CSPM is a pair-matching algorithm that (1) appears to produce results comparable to the Blossom algorithm (which optimally approximates the distribution of partnerships in a partnership market), (2) is practical to use in microsimulations with very large numbers of agents, and (3) is practical to use when many thousands of simulations need to be run in a reasonable amount of time.

### Effect of population size

The finding that incidence declines as population increases when algorithms account for more complex partner matching, is surprising.

This does not appear to be explained by the increasing quality of matches as the partnership market increased. The average distance between the agents in partnerships in the algorithms is approximately 14, 20.5, 26.5, 29.5 and 61 for Blossom, BFPM, CSPM, RKPM and RPM respectively (higher scores mean worse matches). These values do not change much as the number of agents increases. In fact, CSPM has a slightly lower average score for 20,000 agents than 1 million agents. If the larger partnership market resulted in better quality matches, we would expect the average distance to decrease.

Fig 2 depicts what is occurring. During the early stages of the epidemic incidence is lower for a longer period of time, resulting in lower prevalence at any given time in the ten year period of the simulation. When we started simulations in an already mature epidemic (e.g. 10% prevalence), the effect of prevalence being lower with higher numbers of agents disappeared.

A possible explanation for what is happening is that as the number of agents decreases, an infection occurring in a relatively low-risk subgroup of agents (e.g. WSW aged between 45 and 50 years old) has a disproportionately greater effect on the number of infections that will subsequently occur in that subgroup. For example, if there are only 10 WSW aged 45 to 50 in a simulation and one of them, via a poor match or stochastic variation, becomes infected, then 10% of the subgroup is immediately infected. However if in a much bigger population there are 100 agents in this subgroup, then only 1% of this low-risk subgroup is infected. On consequent time steps, the risk of prevalence increasing substantially in this subgroup is much higher for the smaller population. This is particularly the case for Blossom by virtue of the fact that it generally makes better matches than the other algorithms. For CSPM the effect may be due to it clustering agents that are more likely to be paired; even poor matches will be in neighbouring or nearby clusters, and partnering within clusters is accentuated as the population grows.

Further research is needed to understand this phenomenon properly and offer an explanation with confidence.

### Trade-off between complexity and speed

Our results raise a dilemma for STI microsimulation modellers. The advantage of microsimulations over ODE models is that the former can practically account for a much greater number of compartments, i.e. a greater variety of agent characteristics. However, a pair-matching algorithm such as Blossom that best accounts for these characteristics is extremely slow. It can be used effectively only with smaller population sizes or choosing small subgroups of a population (e.g., [20]). However, when the risk of infection is high (e.g. for HPV and gonorrhea), the time horizon is long or the turnover of partnerships is high, modelling with an agent population that is much lower than the real world population of interest may considerably underestimate incidence and prevalence. Although in practice this would be corrected by calibrating the model to real world data points, the calibration process may in turn result in parameter values (i.e., for infection rates) being set far off their real world values, so that the further into the future the model projects, the greater will be the error in its estimates.

Modellers may wish to consider using an algorithm such as CSPM that usually offers a good trade-off between speed and approximation of the distribution of relationships in the population being studied. Ideally a simulation should have a similar number of agents as the population being studied. This is often impractical though, and even where it is practical, the poor quality of data on the distribution of partnerships based on sex, age, sexual orientation and even the role of geographical location, is a much bigger problem.

Of course, if the risk of infection per serodiscordant partnership is low then using a large population for the microsimulation may be unnecessary. The same is true if the simulation begins when an epidemic is already established.

### Limitations

Our analysis has several limitations:

- The DATA strategy is based on sex survey data, with the well-documented problems that this presents [21].
- Our modelling of casual relationships is unsophisticated, and possibly overstates casual sex as one-night stands and understates short-term relationships involving a few sexual encounters. However, our results appear to be robust when accounting for this by greatly reducing the number of casual partnerships.
- We did not model varying the risk of transmission over the course of an infection, for example, the higher transmission risk of HIV during primary infection. This would be particularly interesting to examine in research that extends our work.
- We did not model concurrent relationships, although they might play a significant role in the spread of STIs [22]. This too would be an interesting way to extend our research.
- As noted in the introduction, we did not model birth and mortality, healing, condom use, circumcision and other factors, as including these would confuse the analysis and make it difficult to isolate the role of the partnership market and breakup strategy, and pair-matching algorithm.
- We have not done systematic subgroup analysis, e.g. the effect of the different algorithms on a particular 5-year age group or sexual orientation.
- In our base case analyses, we included a random effect for individual behavior associated with unobserved characteristics of the agents. For some agents, this factor might dominate the influence of the observable characteristics (e.g., age). However, there were no significant differences when we compared these results to analyses that excluded the random effects.
- The base case also included a penalty for previous partners which might not be realistic. Generally, the pairfam dataset allows the identification of partnerships with previous partners. As we summarized recurrent partnership episodes in the parameter estimation, we did not encourage partnerships with previous partners to be consistent with the parameter estimation. This assumption may influence the results, but removing the penalty on previous partnerships in a sensitivity analysis did not produce significantly different results.

In preparing this paper we ran tens of thousands of simulations with the number of agents ranging from 10,000 to as high as 40 million using affordable consumer hardware. The feasibility of this is likely of interest to other modellers; Appendix 1 contains further notes on our implementation.

Further research needs to be done refining the cluster function of CSPM, as well as identifying ideal values of *k* (for RKPM as well) and the number of clusters. A deeper analysis of poor matches, and how frequently to block or allow them is also needed. We also recommend examining the effect of using the CSPM algorithm to model HPV or gonorrhoea in real world populations, using different numbers of agents in the model.

Mismatches in CSPM are more likely to occur between agents in neighbouring clusters. A possible pitfall of CSPM—depending on the domain being studied and how the clustering is implemented—is that the neighbourhood of clusters is arbitrary, and therefore the mismatches will mirror the arbitrariness of the neighbouring clusters. If this is the case, the clusters themselves should be shuffled on each time step of the simulation.

## Conclusion

Microsimulations have become a popular method for the analysis of the spread of STIs and for the evaluation of interventions to alleviate them. While the effects of structural assumptions about pair formation and infectivity are well known for the classical method of ODEs, the analysis of design decisions of microsimulations are less well understood.

Our findings contribute to closing this gap by providing insights into the effect of different matching algorithms for various infection rates. Additionally, we found that there exist fast pair-matching algorithms that provide a practical way for microsimulation modellers to account for more complex sexual behaviour and without limiting the population to a small subgroup. Our findings may also inform reviewers of STI microsimulations about the extent to which the pair matching methodology can influence the results of a model.

## Supporting information

S1 Appendix Computational implementation. Computational implementation of FastSTI.
(PDF)

S2 Appendix Partner market parameters. Age-specific parameter values for entering and leaving the partner market.
(PDF)
