# Supplementary material for: How various design decisions on matching individuals in relationships affect the outcomes of microsimulations of sexually transmitted infection epidemics
Source: PLoS One. 2018 Aug 29;13(8):e0202516. doi: 10.1371/journal.pone.0202516 (PMC6114846; doi:10.1371/journal.pone.0202516)
Supplement: S1 Appendix — Computational implementation of FastSTI. (PDF) [file pone.0202516.s001.pdf]

S1 Appendix. Computational implementation This appendix aims to provide a few pointers to implementing fast microsimulations that can accommodate large numbers of agents. It is mainly aimed at microsimulation modellers who do not have a formal computer science background.

Our model is capable of doing large microsimulations fast. For example, running single-threaded, it can match nearly 600,000 agents per second in a partnership market using the CSPM algorithm on a laptop with an i5 processor running at 2.5GHz. With the agent population set to 40 million, it takes approximately 5.2 hours to run five 10-year simulations in parallel, updated daily, on an Intel Xeon with 20 cores at 2.3Ghz, with 32GB RAM (the RAM was the limiting factor; we could not use more than 5 or 6 threads at a time without running out of memory). Once birth, death, healing, and migration events, as well as a concurrency feature are added, this will slow down, but not substantially, because we expect the time taken for all of these events to increase linearly with the number of agents. On the same machine, using multithreading, we also ran 20,000 simulations of 10,000 agents (10 years, time step equal to a day) in a little more than 6 hours. This is a mean of just over 22 seconds per simulation. Running multiple threads in parallel slows down the execution of a single run because of resource contention. A single-threaded run takes about two seconds.

In a discrete microsimulation of the form described by Algorithm 1, it is the events that consume the most time. The time taken for most events to complete increases linearly with the number of agents. These include ageing, death, healing, select, and breakup events. However, pair-matching requires interaction between agents. The time to run BFPM increases quadratically with the size of the partnership market. The time taken to run the Blossom algorithm is worse: approximately cubic with the number of agents. It takes over 2 hours to match 5,000 agents in a partnership market on an i5 processor running at 2.5GHz (using our default data that would be a simulation containing approximately 1.4 million agents). So simulating 10 years with a 60 day stabilisation period would take about 7,420 hours, or over 300 days. Perhaps waiting this long for the outcome of a physics or chemistry experiment with precise inputs and outputs may be worth it, but waiting more than a day or two for results of a single simulation in the world of STI modelling where nearly every input is a rough approximation seems pointless to us.

Table S1.1 and Figure S1.1 present execution times of simulations using the different pair-matching algorithms.

RPM's time increases linearly, but it does very poor matching. The time for CSPM and RKPM increases loglinearly ($n \log n$, where n is the size of the partnership market) with the number of agents in the partnership market. For a large simulation of 40 million agents to be done in a practical amount of time, it is vital to use an algorithm whose time efficiency is loglinear or better.

Also it is vital to put agents who need to be paired on a particularly time step into a partnership market. This market is almost always a small fraction of the total number of agents. Determining which agents enter the partnership market requires an algorithm with linear efficiency. Pairing the agents intelligently requires an algorithm with at least loglinear efficiency (as far as we can tell). Skipping the partnership market creation, and subsuming it into the pair-matching algorithm is false efficiency, because instead of the pair-matching algorithm executing in time that increases loglinearly with the number of agents in the partnership market, it will

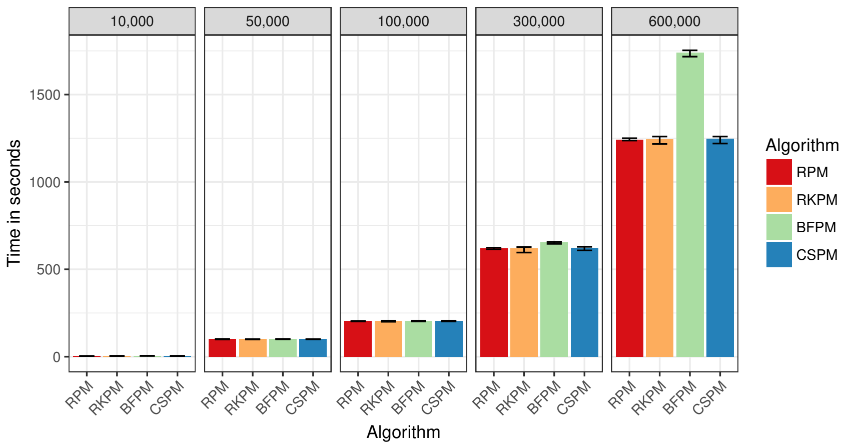

Figure S1.1: **Algorithm execution times**
Average execution time in seconds for each algorithm (except Blossom) when 30 simulations are run in parallel. Note that there are other time-consuming events in a simulation other than pair-matching, and that times are approximate.

Agents	Blossom	BFPM	CSPM	RKPM	RPM
10,000	23	2	2	2	1
100,000	2,960	81	43	42	12
1,000,000	N/A	8,810	633	601	218

Table S1.1: Time in seconds for a single execution on an Intel Xeon i7 2.3GHZ without any other significant load on the machine. Note that there are other time-consuming events in a simulation other than pair-matching, and that times are approximate.

execute with time that increases loglinearly with the total number of agents in the simulation, a much larger number.

In computer science, we describe algorithms whose efficiency increases linearly, loglinearly, quadratically and cubicly with a dataset with n elements as $O(n)$, $O(n \log n)$, $O(n^2)$ and $O(n^3)$ respectively. We call this Big O notation and the preceding list is ordered from fastest to slowest efficiency [1]. When optimising code, changing a time-consuming algorithm to one whose Big O efficiency is better is usually more productive than tinkering with the implementation details of a fundamentally inefficient algorithm. For example, no matter how much we optimise our implementation of the BFPM algorithm it will not be as fast as an unoptimised competent implementation of the CSPM algorithm. This is a necessarily simplified discussion of Big O. For further details see one of many textbooks on the subject, eg. [1].

From our knowledge of the field and discussions with other modellers, it appears some leading microsimulation models are coded in interpreted languages such as R, Visual Basic or Python. While microsimulations coded in these languages have many uses, there is unfortunately little prospect

[1] This is a very simplified discussion and intentionally omits the point that Big O notation are sets to which algorithms belong, or that a less efficient set includes all the algorithms in more efficient sets. These details are simply unnecessary here.

that these languages can develop microsimulations that can manage the kind of loads described in this paper. For heavy-duty simulation it is vital to use a programming language that is either compiled directly into native machine code (e.g. C, C++, Rust, Fortran, Common Lisp, Swift, Go) or the byte code of a virtual machine (e.g. Java, Clojure, Scala).

Our microsimulation, which we call FastSTI, is open source and, we hope, well commented and easy to read. Modellers are encouraged to download it, adapt it for their needs, report bugs to us, and send us queries.

References

[1] Knuth DE. The Art of Computer Programming, Volume 1: (3rd Ed.) Fundamental Algorithms. Redwood City, CA, USA: Addison Wesley Longman Publishing Co., Inc.; 1997.
