# Supplementary material for: How various design decisions on matching individuals in relationships affect the outcomes of microsimulations of sexually transmitted infection epidemics
Source: PLoS One. 2018 Aug 29;13(8):e0202516. doi: 10.1371/journal.pone.0202516 (PMC6114846; doi:10.1371/journal.pone.0202516)
Supplement: S2 Appendix — Age-specific parameter values for entering and leaving the partner market. (PDF) [file pone.0202516.s002.pdf]

S2 Appendix. Partner market parameters As stated in the Materials and Methods section, the input parameters have been derived from the PAIRFAM study. The data includes eight waves of observations from 2008 to 2016 for three age cohorts born 1971-73, 1981-83 and 1991-93. The data is freely available at `http://www.pairfam.de/` after becoming a registered user.

While the probabilities for breakups and entering the partnership market were calulated directly from the data, the probability of casual sex was smoothed using a cubic-spline regression with age as the only explanatory variable and using the predicted values for the complete age-range of 12 to 50. See Table S2.1 and Figure S2.1.

Age	Casual Sex		Long-term relationships			
	Enter partnership market		Enter partnership market		Breakup	
	Female	Male	Male	Female	Male	Female
12	0.00914	0.00000	0.00002	0.00003	0.00069	0.00044
13	0.01194	0.00000	0.00011	0.00015	0.00097	0.00067
14	0.01474	0.00453	0.00024	0.00035	0.00122	0.00085
15	0.01753	0.01432	0.00040	0.00063	0.00139	0.00093
16	0.02030	0.02418	0.00055	0.00089	0.00136	0.00090
17	0.02302	0.03392	0.00066	0.00106	0.00117	0.00079
18	0.02564	0.04307	0.00072	0.00115	0.00095	0.00066
19	0.02814	0.05110	0.00075	0.00118	0.00078	0.00055
20	0.03056	0.05744	0.00076	0.00116	0.00065	0.00045
21	0.03291	0.06168	0.00076	0.00112	0.00055	0.00038
22	0.03519	0.06356	0.00076	0.00106	0.00048	0.00032
23	0.03741	0.06327	0.00077	0.00102	0.00042	0.00027
24	0.03956	0.06130	0.00079	0.00103	0.00039	0.00025
25	0.04160	0.05818	0.00081	0.00107	0.00037	0.00025
26	0.04349	0.05447	0.00083	0.00110	0.00035	0.00024
27	0.04517	0.05077	0.00083	0.00111	0.00031	0.00023
28	0.04658	0.04765	0.00083	0.00108	0.00026	0.00020
29	0.04773	0.04543	0.00081	0.00101	0.00022	0.00017
30	0.04859	0.04426	0.00078	0.00093	0.00020	0.00015
31	0.04907	0.04389	0.00074	0.00084	0.00018	0.00014
32	0.04905	0.04400	0.00068	0.00076	0.00015	0.00012
33	0.04846	0.04436	0.00062	0.00069	0.00013	0.00011
34	0.04725	0.04484	0.00057	0.00064	0.00012	0.00011
35	0.04555	0.04538	0.00056	0.00061	0.00012	0.00012
36	0.04352	0.04595	0.00056	0.00061	0.00014	0.00012
37	0.04132	0.04646	0.00057	0.00061	0.00014	0.00012
38	0.03910	0.04681	0.00059	0.00060	0.00013	0.00011
39	0.03696	0.04677	0.00060	0.00059	0.00012	0.00010
40	0.03496	0.04616	0.00061	0.00058	0.00012	0.00010
41	0.03315	0.04487	0.00062	0.00059	0.00012	0.00011
42	0.03153	0.04289	0.00064	0.00062	0.00013	0.00012
43	0.03005	0.04036	0.00067	0.00065	0.00014	0.00012
44	0.02865	0.03745	0.00071	0.00069	0.00015	0.00013
45	0.02728	0.03439	0.00075	0.00073	0.00016	0.00013
46	0.02593	0.03130	0.00078	0.00077	0.00016	0.00014
47	0.02458	0.02820	0.00082	0.00081	0.00017	0.00015
48	0.02323	0.02511	0.00087	0.00084	0.00018	0.00015
49	0.02188	0.02201	0.00091	0.00088	0.00018	0.00016
50	0.02053	0.01891	0.00095	0.00093	0.00019	0.00016

Table S2.1: Daily probabilities of entering the partnership market for agents who are single, or breaking up for agents in relationships.

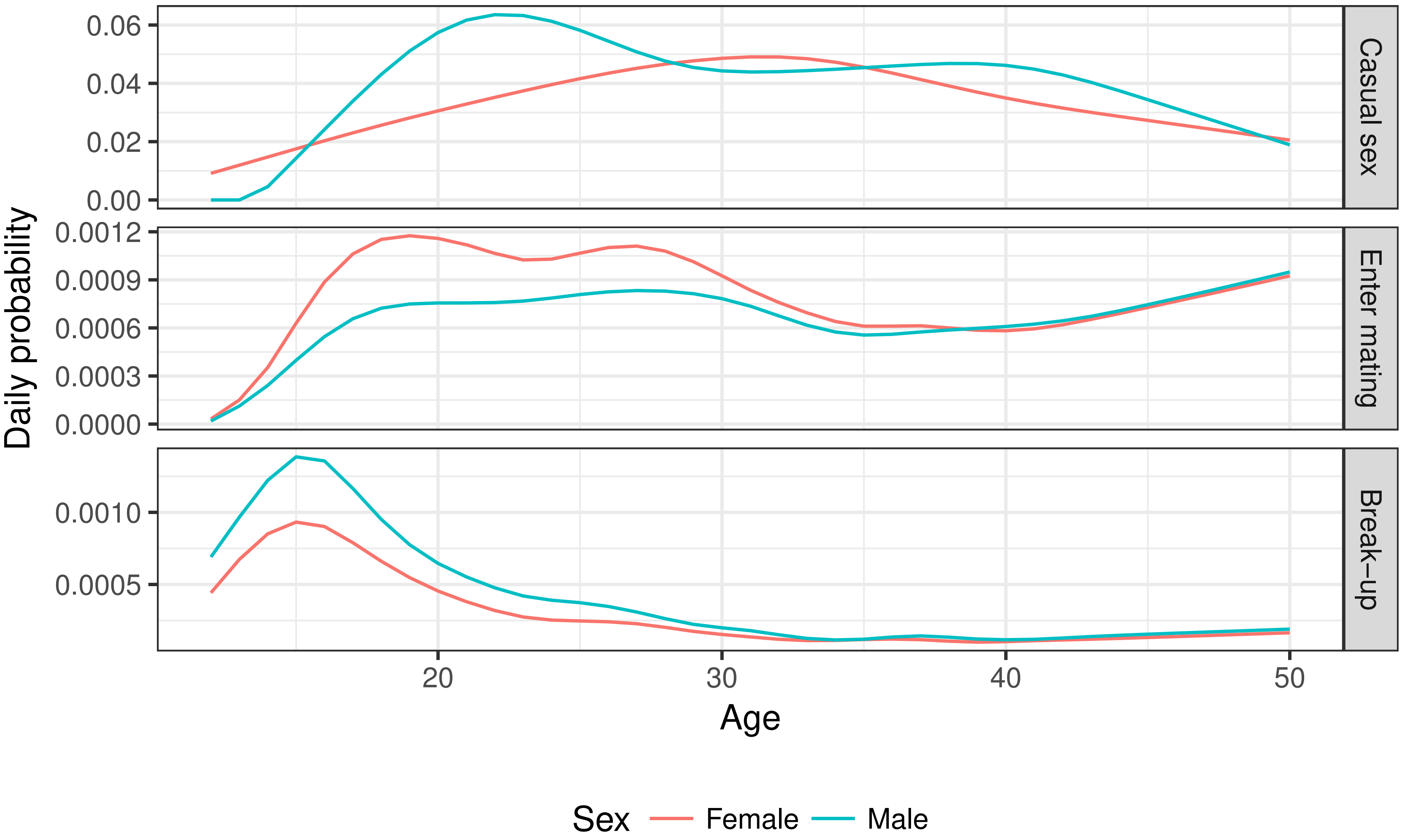

Figure S2.1: **Partnership market probabilities**
Daily probabilities (by age and sex) of entering the partnership market for agents who are single, or breaking up for agents in relationships.
